# Ab Initio Study of the Large Amplitude Motions of Various Monosubstituted Isotopologues of Methylamine (CH_3_-NH_2_)

**DOI:** 10.3389/fchem.2021.751203

**Published:** 2021-09-23

**Authors:** Muneerah Mogren Al-Mogren, María Luisa Senent

**Affiliations:** ^1^ Chemistry Department, Faculty of Science, King Saud University, Riyadh, Saudi Arabia; ^2^ Departamento de Química y Física Teóricas, Instituto de Estructura de La Materia, IEM-CSIC, Madrid, Spain

**Keywords:** methylamine, LAM, torsion, wagging, ^13^CH_3_NH_2_, CH_3_^15^NH_2_, CH_3_NHD, CH_2_DNH_2_

## Abstract

CCSD(T)-F12 theory is applied to determine electronic ground state spectroscopic parameters of various isotopologues of methylamine (CH_3_-NH_2_) containing cosmological abundant elements, such as D, ^13^C and ^15^N. Special attention is given to the far infrared region. The studied isotopologues can be classified in the G_12_, G_6_ and G_4_ molecular symmetry groups. The rotational and centrifugal distortion constants and the anharmonic fundamentals are determined using second order perturbation theory. Fermi displacements of the vibrational bands are predicted. The low vibrational energy levels corresponding to the large amplitude motions are determine variationally using a flexible three-dimensional model depending on the NH_2_ bending and wagging and the CH_3_ torsional coordinates. The model has been defined assuming that, in the amine group, the bending and the wagging modes interact strongly. The vibrational levels split into six components corresponding to the six minima of the potential energy surface. The accuracy of the kinetic energy parameters has an important effect on the energies. Strong interactions among the large amplitude motions are observed. Isotopic effects are relevant for the deuterated species.

## Introduction

Methylamine (CH_3_-NH_2_) plays important roles in the gas phase chemistry in the terrestrial and extraterrestrial atmospheres. The presence in the Earth’s atmosphere has both natural and anthropogenic causes (Ge et al., 2011). In air quality studies, it is considered to be a Volatile Organic Compound (VOC) that can be a precursor of secondary organic aerosols (SOA) in the presence of glyoxal (De Haan et al., 2009). In 1974, it was detected in the interstellar medium and it is contemplated as a relatively abundant species (Kaifu et al., 1974) (Fourikis et al., 1974). Recent studies consider it a precursor of glycine and a building block of life (Ohoshi et al., 2019). Recently, methylamine has been detected in the quasar PKS 1830-211 (Muller et al., 2011) and together with other simple N-bearing species, it has been observed in the hot cores NGC 6334I MM1-3 (Bøgelund et al., 2019). Fourikis et al. (1977) have reported the probable detection of deuterated methylamine (CH_3_NHD) in Sgr B2.

The aim of the present work is the theoretical study of probably detectable methylamine isotopologues. Monosubstituted isotopologues were detected for many astrophysical molecules such as dimethyl-ether and methyl-formate as it is described in the references provided by the papers of Fernández et al. (2019) and Gámez et al. (2019). In a recent study of the methylamine main isotopologue, highly correlated ab initio methods were employed to simulate the far infrared spectra (Senent 2018). The low-lying vibrational energy levels in and their tunneling splitting components were computed, providing relevant information for rotational spectrum assignments, which are mandatory for the detection using radio-astronomy. Very accurate results were obtained by comparing with previous experimental data. A detailed review of previous theoretical and experimental works can be found in Senent (2018).

The motivation of many previous studies of methylamine concerns more to the peculiar molecular structure than to its applications (Hamada et al., 1982) (Ohashi & Hougen 1987), because it is contemplated as a prototype small non-rigid molecule where two interacting large amplitude motions, the torsion of the methyl group and the NH_2_ wagging, govern its internal dynamics (Ohashi & Hougen 1987) (Kreglewski 1978, 1989) (Ohashi & Toriyama 1994 (Kleiner and Hougen, 2015). High resolution rovibrational spectra have been measured for the ground and various excited vibrational states, given a special attention to the far infrared region (Belorgeot et al., 1982) (Diallo et al., 1985) (Ohashi et al., 1987, 1988, 1989, 1992) (Ilyushin et al., 2005) (Kreglewski & Winther 1992) (Kreglewski & Wlodarczak 1992) (Motiyenko et al., 2014) (Nguyen et al., 2021) (Dawadi et al., 2013a; 2013b).

Whereas publications about the methylamine main isotopologue are recurrent, less studies attend to other isotopic species. The microwave spectrum of the monosubstituted species CH_3_NHD (Ohashi et al., 1991), CH_2_DNH_2_ (Tamagake & Tsuboi 1974), and ^13^CH_3_NH_2_ (Motiyenko et al., 2016), and the deuterated species, CH_3_ND_2_, CD_3_NH_2_, CD_3_ND_2_ (Lide 1954) (Sastry 1960) (Takagi & Kojima 1971) (Kreglewski et al., 1990a; 1990b) were measured and assigned. The infrared absorption spectrum of ^15^N-methylamine was inspected in the gas phase (Hirakawa et al., 1972). Mass resolved excitation spectroscopy and ab initio calculations were employed to analyze the low-lying excited states of CH_3_NH_2_, CH_3_NH2, CD_3_NH_2_, CH_3_ND_2_, and CD_3_ND2 (Taylor & Bernstein 1995). The A←X excitation spectra of six different deuterated isotopologues including the CH_3_NHD monosubstituted species, were explored (Park et al., 2006).

Previous studies devoted to n-methyl amines describe theoretical techniques and symmetry concepts useful for the present work (Senent & Smeyers 1996) (Smeyers et al., 1996, 1998) (Senent 2018)On the basis of previous ab initio results (Senent 2018), performed using explicitly correlated coupled cluster theory, CCSD(T)-F12 (Adlet et al., 2007) (Knizia et al., 2009), in this new paper, we attend to several monosubstituted isotopologues containing abundant cosmological elements. Although, to our knowledge, a unique isotopologue CH_3_NHD has been probably detected (Fourikis et al., 1977), other species are considered to be detectable species. Four isotopic species, ^13^CH_3_NH_2_, CH_3_^15^NH_2_, CH_3_NHD, and CH_2_DNH_2_, are studied and compared with the main isotopologue for predicting theoretically isotopic shifts. Recently, interstellar amines and their fragments have been studied using quantum-chemical computations (Salta et al., 2020) (Puzzarini et al., 2020).

An earliest CCSD(T)-F12 three-dimensional potential energy surface is revisited in the present work (Senent 2018) because it is mass independent. It is employed for constructing mass dependent effective potential energy surfaces for the different isotopologues. The surfaces present six minima separated by relatively low potential energy barriers. If the minimum interconversion is taken into consideration, the most abundant isotopologue can be classified in the G_12_ molecular symmetry group (Ohashi & Hougen 1987). The isotopic substitutions carry out changes in the symmetry. Details concerning the followed procedure can be found in our previous paper devoted to the acetone isotopologues (Dalbouha et al., 2021). The effective surfaces allow to construct Hamiltonians depending on three interacting coordinates, two interacting large amplitude motions, the NH_2_ wagging and the CH_3_ torsion, and the HNH bending. Then, both the bending and wagging of the amine group are treated together. The final levels are computed variationally.

## Results and Discussion

### Electronic Structure Calculations

The theoretical study of methylamine isotopologues was started from the results of a previous work devoted to the main isotopologue CH_3_NH_2_ (Senent 2018). In this earlier paper, the structural parameters of the minimum energy structure and a three-dimensional ab initio potential energy surface (3D-PES) were computed using explicitly correlated coupled cluster theory with single and double substitutions augmented by a perturbative treatment of triple excitations (CCSD(T)-F12b) (Adlet et al., 2007) (Knizia et al., 2009) using the MOLPRO package default options (Werner et al., 2012). The procedure was applied in connection with the AVTZ-F12 basis set, which contains the Dunning’s type aug-cc-pVTZ atomic orbitals (AVTZ) (Kendall et al., 1992) and the corresponding functions for the density fitting and the resolutions of the identity. These previous computed data are mass independent properties that can be used for the different isotopic species.

To determine the core-valence electron correlation effects on the rotational constants, the structure was optimized using CCSD(T) (coupled-cluster theory with single and double substitutions, augmented by a perturbative treatment of triple excitations) (Hampel et al., 1992) and the cc-pCVTZ basis set (CVTZ) (Woon and Dunning Jr 1995).

The full-dimensional anharmonic force field and the vibrational corrections of the potential energy surface are mass dependent properties that must be computed for each isotopologue. For this reason, new electronic structure calculations have been performed in the present work. That properties were determined using second order Möller-Plesset theory (MP2) (Møller & Plesset 1934) implemented in GAUSSIAN (Frisch et al., 2016). Anharmonic force fields allow obtain spectroscopic properties using second order perturbation theory (VPT2) (Barone 2005) (Bloino et al., 2012). The vibrationally corrected surfaces were employed to construct Hamiltonians for the isotopologues. The energy levels corresponding to the large amplitude vibrations and to the HNH bending mode were computed using a variational procedure implemented in ENEDIM (Senent 1998a; 1998b, 2001).

### The Symmetry of the Isotopologues

The main isotopologue, as well as ^13^CH_3_NH_2_ and CH_3_^15^NH_2_, can be classified in the G_12_ molecular symmetry group (MSG) (Ohashi & Hougen 1987) and in the C_s_ point group. However, the H →D substitution carries out changes in the symmetry properties. CH_3_NDH must be classified in the C_1_ point group and in the G_6_ MSG, due to the absence of the symmetry plane. In CDH_2_NH_2,_ the D atom can replace the in-plane H atom (C_s_-CDH_2_NH_2_) or one out-of plane H atom (C_1_-CDH_2_NH_2_). If VPT2 is applied and a unique minimum is considered, the molecule is assumed to be semi-rigid and all the vibrations are described as small displacements around the equilibrium. Two different point groups C_1_ and C_s_ are used. However, if the internal rotation is taken into account, C_s_-CDH_2_NH_2_ and C_1_-CDH_2_NH_2_ represent different minima of the same potential energy surface and they can be inter-converted. Then, both are classified in the same G_4_ MSG.

The G_12_ MSG contains six irreducible representations, four non-degenerate, A_1_, A_2_, B_1_ and B_2_, and two double-degenerate E_1_ and E_2_. The G_6_ MSG contains three irreducible representations, two non-degenerate, A_1_, and A_2_, and one double-degenerate E. The G_4_ MSG contains four non-degenerate irreducible representations, A_1_, A_2_, B_1_, and B_2_.

### Rovibrational Parameters

**FIGURE 1:**
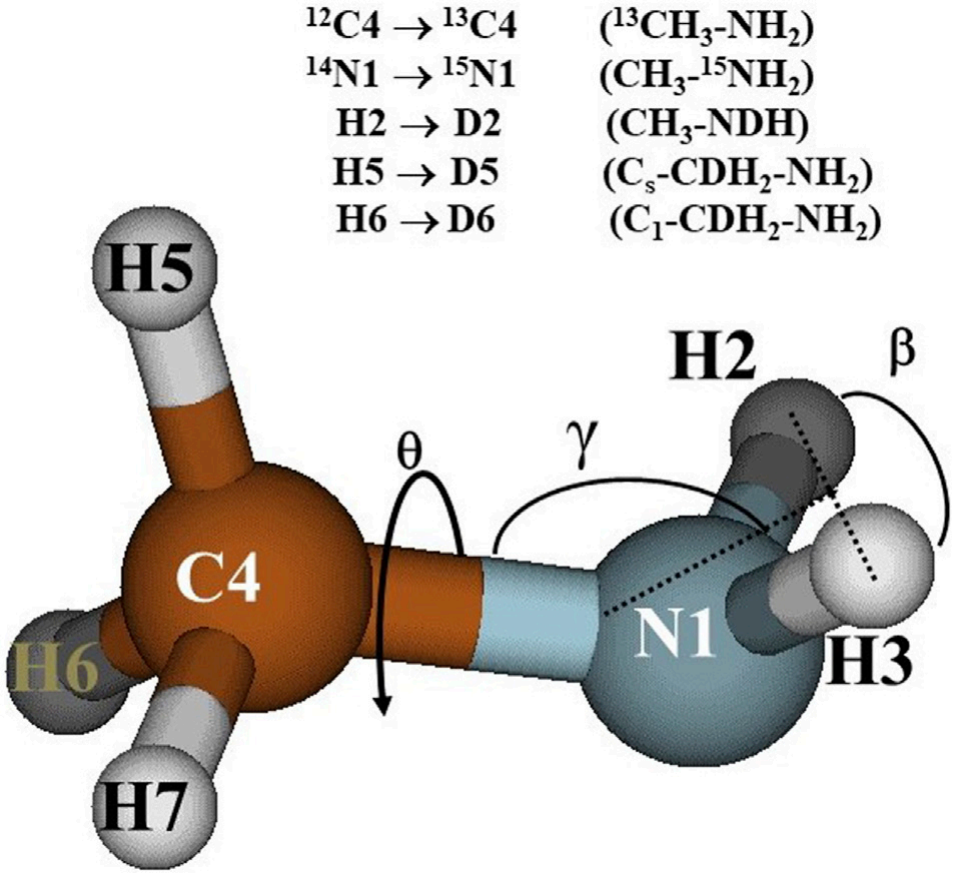
Methylamine equilibrium structure.

In the earlier paper (Senent 2018), the CCSD(T)-F12/AVTZ structural parameters of the methylamine equilibrium geometry, are detailed. The structure is shown in Figure 1, that helps to understand the atom labelling and the isotopic substitutions.

For all the isotopologues, the vibrational ground state rotational constants shown in Table 1, were computed from the CCSD(T)-F12 equilibrium rotational constants using the following equation:

B0= Be (CCSD(T)-F12/AVTZ-F12) + △Be(CCSD(T)/CVTZ)core+△Bvib(MP2/AVTZ) (1)

**TABLE 1:** Rotational constants (in MHz).

	CH_3_NH_2_ (Cs)		^13^CH_3_NH_2_ (C_s_)		CH_3_^15^NH_2_ (C_s_)
A_e_	103,855.395		103,851.612		103,751.743
B_e_	22,803.133		22,267.093		22,292.683
C_e_	21,926.385		21,430.485		21,458.454
	Senent (2018a)	Ilyushin et al. (2005)	*This work*	Motiyenko et al. (2016)	*This work*
A_0_	103,067.129	103,155.749	103,110.685	103,158.312	103,012.808
B_0_	22,588.290	22,608.305	22,061.169	22,080.995	22,086.384
C_0_	21,710.496	21,730.428	21,221.438	21,242.856	21,248.664
					
	CH_3_NDH (C1)		C_s_- CDH_2_NH_2_/C_1_- CDH_2_NH_2_		
A_e_	90,053.981		86,604.055/87,207.318	
B_e_	21,528.245		20,720.150/21,422.590		
C_e_	20,266.914		20,649.766/20,082.096		
	Calc	Ohashi et al. (1991)	*This work*		
A_0_	89,438.271	89,523.02	86,037.713/86,570.015		
B_0_	21,334.679	21,333.37	20,526.133/21,225.188		
C_0_	20,072.203	20,118.07	20,455.807/19,894.077		

Here, ΔB_e_^core^ collects the core-valence electron correlation effects on the equilibrium structure and ΔB^vib^ represents the vibrational contribution derived from the second order perturbation theory (VPT2) *α*_*r*_^*i*^ vibration-rotation interaction parameters. These last were determined using the MP2/AVTZ cubic force fields and vibrational second order perturbation theory. ΔB_e_^core^ was determined from the CCSD(T)/CVTZ parameters B_e_ (CV) and B_e_(V), calculated correlating both core and valence electrons (CV) or just the valence electrons (V) in the post-SCF process. Then:

$$\Delta {\text{B}}_{\text{e}}^{\text{core}}{\;=\text{ B}}_{\text{e}}\left(\text{CV}\right){\;-\text{ B}}_{\text{e}}\left(\text{V}\right)$$ (2)

This approximation has been corroborated in previous studies of other non-rigid molecules providing really accurate parameters, whose deviations with respect available experimental data, represent few MHz (Boussesi et al., 2016) (Dalbouha et al., 2016, 2021). In Table 1, the computed rotational constants of CH_3_NH_2_, ^13^CH_3_NH_2,_ and CH_3_NDH are compared with available experimental parameters (Ilyushin et al., 2005) (Motiyenko et al., 2016) (Ohashi et al., 1991). The MP2/AVTZ quartic centrifugal distortion constants corresponding to the asymmetrically reduced Hamiltonian, are shown in Table 2 where they are compared with previous experimental data (Ilyushin et al., 2005) (Motiyenko et al., 2016) (Ohashi et al., 1991). Disagreements between experimental and computed data can be correlated with the level of ab initio calculations used to compute the anharmonic force field. In addition, in methyl amine, the interaction between the internal and global rotation causes deviations. Isotopic shifts are more reliable.

**TABLE 2:** MP2/AVTZ quartic (in KHz) centrifugal distortion constants^a^ computed using the MP2/AVTZ cubic force fields.

	CH_3_NH_2_ (C_s_)		^13^CH_3_NH_2_ (C_s_)		CH_3_^15^NH_2_ (C_s_)
	*This work*	Ilyushin et al. (2005)	*This work*	Motiyenko et al. (2016)	*This work*
Δ_J_	38.7083	39.4506(18)	37.3369	38.06084(18)	37.3734
Δ_K_	610.0394	701.049(24)	641.8328	706.766(12)	643.7290
Δ_JK_	172.7131	170.983(15)	161.3963	166.8639(18)	161.0483
δ_J_	1.6377	1.75679(17)	1.5367	1.660,274(31)	1.5536
δ_K_	−217.5746	−337.78(14)	−226.9603	−322.295(13)	−223.0754

	CH_3_NDH (C_1_)		C_s_- CDH_2_NH_2_/C_1_- CDH_2_NH_2_	
	*This work*	Ohashi et al. (1991)	*This work*	
Δ_J_	33.6435	33.22(93)	32.5197/32.9086	
Δ_K_	454.6168	682.0(13)	487.9419/392.1165	
Δ_JK_	154.6421	128.1(94)	142.1828/167.5383	
δ_J_	2.0344		0.1886/2.3911	
δ_K_	−88.5972		−6,125.3195/19.9638	

The anharmonic fundamental frequencies shown in Table 3, were computed using VPT2 theory (Barone 2005) (Bloino et al., 2012) implemented in Gaussian (Frisch et al., 2016) and the MP2/AVTZ force fields. The modes are ordered following the criteria used for the main isotopologue that helps to make visible the isotopic shifts. Although VPT2 does not represent the proper treatment for the study of the vibrations responsible for the non-rigidity, it provides a good description of the mid- and near-infrared regions and a useful first description of the far-infrared region. In addition, it allows predict possible band displacements due to Fermi resonances. VPT2 theory ignores the inter-conversion of minima and treats the molecule as a semi-rigid species with a single minimum. If the existence of a single minimum is assumed, the resulting VPT2 properties are different for C_s_-CDH_2_NH_2_ than for C_1_-CDH_2_NH_2_.

**TABLE 3:** Anharmonic fundamental frequencies (in cm^−1^) calculated in this work and measured in previous experiments in the gas phase^a^.

		**CH_3_NH^2^**		**^13^CH_3_NH_2_**	**CH_3_^15^NH_2_**	
**Mode**	**assign.^b^**	**Senent (2018)**	**Shimanouchi (1972)**	***This work***	***This work***	**Hirakawa et al., 1972**
1	NH_2_ st	3,388	3,361	3,385	3,380	3,354.5
			3,360			
2	CH_3_ st	3,001	2,961	**2,989**	**3,010**	2,961.2
			2,960			
3	CH_3_ st	**2,931**	2,820	**2,909**	**2,916**	2,820
			2,820			
4	NH_2_ b	**1,610**	1,623	**1,639**	**1,635**	1,618.7
5	CH_3_ b	1,481	1,473	1,476	1,478	1,473.6
6	CH_3_ b	1,453	1,430	1,426	1,433	1,430.4
7	HCN b	1,146	1,130	1,127	1,131	1,126.2
8	NC st	1,055	1,044	1,032	1,037	1,031.7
9	NH_2_ wag	781	780	787	783	775.8
10	NH_2_ st	3,464	3,427	3,462	3,453	3,415
11	CH_3_ st	3,034	2,985	3,021	3,031	2,985
12	CH_3_ b	1,481	1485c	1,495	1,495	1,485
13	HNC b	1,315		**1,292**	**1,296**	
14	CH_3_ b	971		965	966	
15	CH_3_ tor	288	268	274	274	
			264.58204^d^			
			264.58279^d^			
			264.58314^e^			
			264.58337^f^			
		**CH**_**3**_ **NDH**	**Cs-CDH**_**2**_ **NH**_**2**_ **/C1-CDH**_**2**_ **NH**_**2**_			
**mode**	**assign.^b^**	**This work**	**This work**			
1	NH_2_ st	2,528	3,384/3,385			
2	CH_3_ st	3,000	**2,998**/**2,898**			
3	CH_3_ st	**2,915**	**2,160**/**2,228**			
4	NH_2_ b	**1,461**	1,605/**1,654**			
5	CH_3_ b	1,478	1,458/1,476			
6	CH_3_ b	1,432	1,337/1,324			
7	HCN b	**1,152**	1,078/1,062			
8	NC st	1,038	920/1,046			
9	NH_2_ wag	691	767/780			
10	NH_2_ st	3,422	3,462/3,462			
11	CH_3_ st	3,032	3,025/3,010			
12	CH_3_ b	1,496	1,373/**1,356**			
13	HNC b	1,219	**1,246**/1,228			
14	CH_3_ b	878	937/845			
15	CH_3_ tor	247	265/262			

a) Emphasized in bold the transitions displaced by Fermi resonances.

b) st = stretching; b = bending; w = wagging; tor = torsion.

c) Hirakawa et al., 1972; d) Ohashi et al., 1989; e) Kreglewski & Wlodarczak 1992; f) Gulaczyk et al., 2017.

The frequencies corresponding to the main isotopologue are compared with experimental data measured in the gas phase (Ohashi et al., 1989) (Kreglewski & Wlodarczak 1992) (Gulaczyk et al., 2017) (Hirakawa et al., 1972) [58]. Previous results are available for CH_3_^15^NH_2_ (Hirakawa et al., 1972). Deviation for several modes are significant, whereas the isotopic shits computed at the MP2 level of theory are reliable.

In Table 3, emphasized in bold, are the fundamental frequencies for which resonances can be relevant. Displacements due to the Fermi interactions were found to be relevant for the ν_3_ fundamental (CH_3_ st), that interacts with two overtones (2ν_6_ and 2ν_12_). The NH_2_ bending fundamental is predicted to interact strongly with the NH_2_ wagging overtone. Since both amine vibrations behave as inseparable modes, the variational model used for exploring the far infrared region, includes explicitly the bending coordinate.

### The far Infrared Spectrum

As was assumed in the previous paper devoted to the main isotopologue (Senent 2018), the low-lying vibrational energy levels corresponding to the two large amplitude motions, the methyl torsion (θ) and the amine NH_2_ wagging (α) can be determined by solving variationally a three-dimensional Hamiltonian where a third coordinate, the HNH bending angle (β), is considered to be an independent variable. The Hamiltonian obeys the formula:

$$H\left(\text{β},\alpha ,\theta \right)=-\overset{3}{\underset{i=1}{\sum }}\overset{3}{\underset{j=1}{\sum }}\left(\frac{\partial }{\partial q_{i}}\right)B_{q_{i}q_{j}}\left(\text{β},\alpha ,\theta \right)\left(\frac{\partial }{\partial q_{j}}\right)+V^{eff}\left(\text{β},\alpha ,\theta \right)$$ (3)

This Hamiltonian was defined by taking into consideration the predictions of the test of resonances described in the previous section and in the previous paper (Senent 2018). Significant interactions between the NH_2_ bending and wagging vibrational modes were predicted. This fact suggests the prerequisite of a 3D-model. In Eq. 3, $B_{qiqj}$ and V^eff^ represent the kinetic energy parameters and the effective potential defined as the sum of three contributions:

$${\text{V}}^{\text{eff}}\left(\beta \text{,}\alpha \text{,}\theta \right)\;=\text{V}\left(\beta \text{,}\alpha \text{,}\theta \right)\;+\text{ V}\prime \left(\beta \text{,}\alpha \text{,}\theta \right){+\text{V}}^{\text{ZPVE}}\left(\beta \text{,}\alpha \text{,}\theta \right)$$ (4)

Here, V( $\text{β},\alpha ,\theta_{\;}$ ) is the mass independent ab initio three-dimensional potential energy surface; V’( $\text{β},\alpha ,\theta_{\;}$ ) and V^ZPVE^( $\text{β},\alpha ,\theta_{\;}$ ) represent the Podolsky pseudopotential and the zero point vibrational energy correction (Dalbouha et al., 2021). The two last contributions must be computed for all the isotopologues because they are mass dependent properties. β, α, and θ, the HNH bending, the NH2 wagging and the torsional coordinates, are defined using curvilinear internal coordinates:

$$\begin{array}{c} \beta {=\text{HNH}-\text{HNH}}^{\text{e}}\\ \alpha =180\text{.0}-\gamma \\ \theta =\left(\text{H}5\text{C}4\text{N}1\text{X}+\text{H6C4N1X}+\text{H7C4N1X }-2\Pi \right)/3 \end{array}$$ (5)

HNH^e^ is the value of the HNH bending angle corresponding to the equilibrium geometry; γ represents the angle between the C-N bond and the HNH plane (see Figure 1); X denotes a ghost atom lying in the HNH plane perpendicular to the HNH angle bisector. The set of internal coordinates were chosen taking into consideration the procedure for the determination of the 3D-PES which demands a partial optimization of the geometry. Three internal coordinates, NHN, γ and H5C4N1X distinguish the selected conformations whereas twelve “dependent coordinates” are allowed to be relaxed in all the structures.

The ab initio three-dimensional potential energy surface, V( $\text{β},\alpha ,\theta_{\;}$ ), was computed for the study of the main isotopologue (Senent 2018). It was constructed using the CCSD(T)-F12/AVTZ energies of 131 geometries defined for selected values of the independent coordinates that were fitted to the following series:

$$V\left(\beta ,\alpha ,\theta \right)=\;\sum_{K,L,M}A_{KML}^{CC}\beta^{K}\cos L\alpha \cos6M\theta +A_{KML}^{SS}\beta^{K}\sin L\alpha \sin3\left(2M+1\right)\theta$$ (6)

This analytical expression transforms as the totally symmetric representation of the G_12_ MSG. Formally identical expressions can be employed for V’, V^ZPVE^, V^eff^( $\text{β},\alpha ,\theta_{\;}$ ) and the diagonal kinetic energy parameters B_qiqi_ of the main isotopologue, ^13^CH_3_NH_2_ and CH_3_^15^NH_2_. However, since the H →D substitution carries out symmetry changes, the effective potential V^eff^( $\text{β},\alpha ,\theta_{\;}$ ) and the diagonal kinetic parameters must be expressed using less-symmetric analytical expressions. For CH_3_NDH (G_6_):

$$V^{eff}\left(\beta ,\alpha ,\theta \right)=\;\sum_{K,L,M}A_{KML}^{CC}\beta^{K}\cos L\alpha \cos3M\theta +A_{KML}^{SS}\beta^{K}\sin L\alpha \sin3M\theta$$ (7)

and for CDH_2_NH_2_ (G_4_)

$$V^{eff}\left(\beta ,\alpha ,\theta \right)=\;\sum_{K,L,M}A_{KML}^{CC}\beta^{K}\cos L\alpha \cos2M\theta +A_{KML}^{SS}\beta^{K}\sin L\alpha \sin\left(2M+1\right)\theta$$ (8)

To construct the effective potential using Eq. 4, two mass-dependent properties V′ and V^ZPVE^ must be computed for all the isotopologues and for all the geometries. The V’ pseudopotential is very small. However, V^ZPVE^ has important effects on the levels. It was determined within the harmonic approximation at the MP2/AVTZ level of theory. To obtain the mass-dependent properties of the low-symmetry varieties, more than 131 geometries and more than 131 sets of harmonic frequencies need to be computed. For example, in the case of CDH_2_NH_2_, 131x3 geometries are required because the three hydrogen atoms of the methyl group are not identical.

**FIGURE 2:**
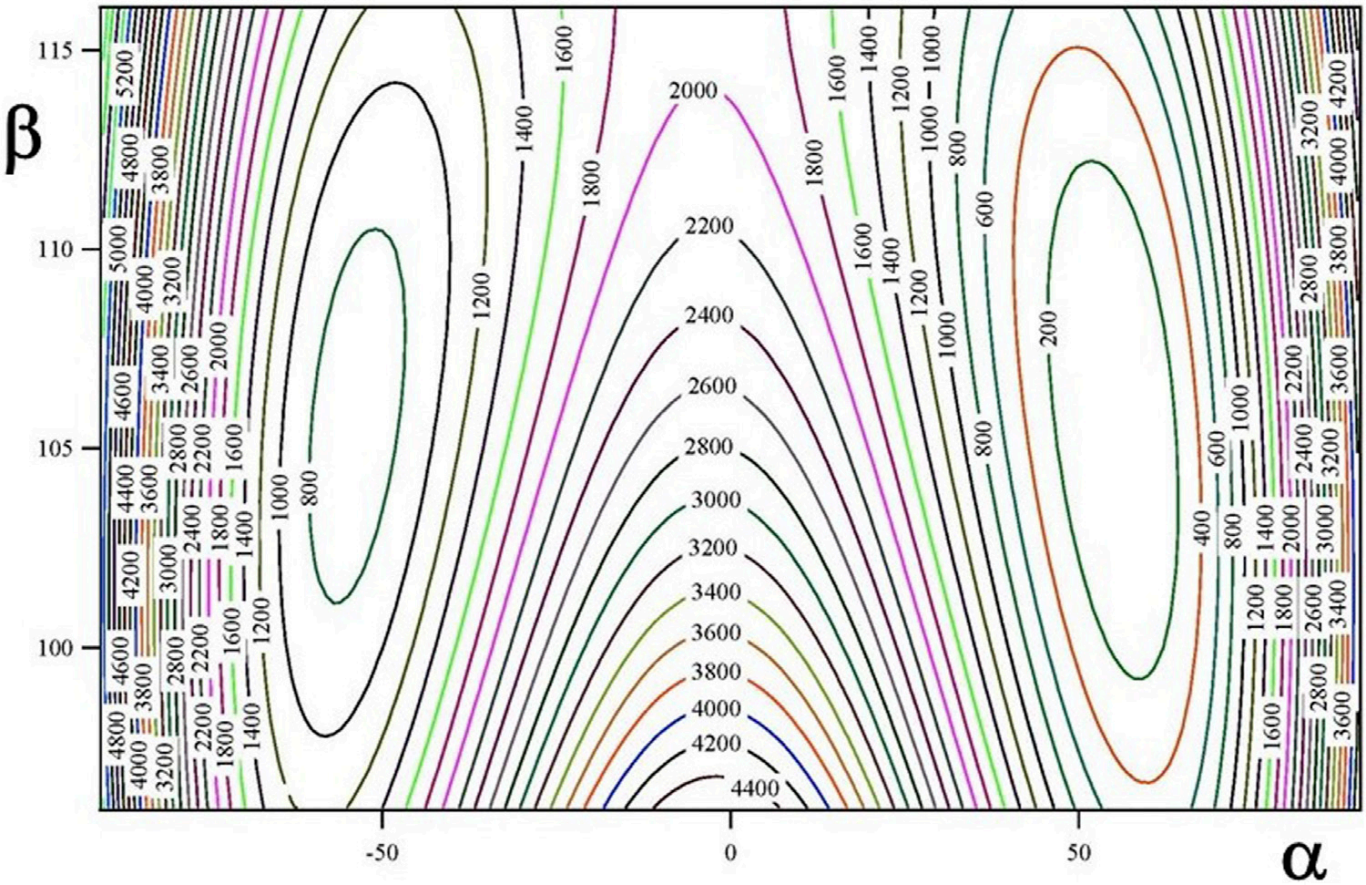
V^eff^ (α, β; θ = 270°) two dimensional potential energy surface (in cm^−1^).

**FIGURE 3:**
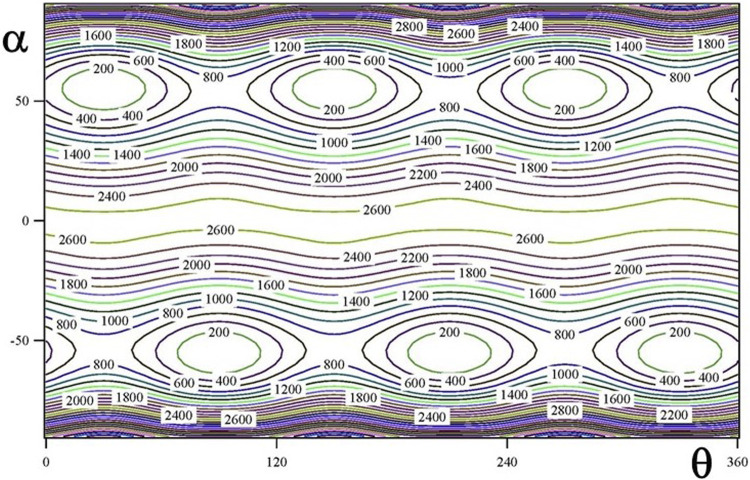
V^eff^ (α, θ; β = 106°) two dimensional potential energy surface (in cm^−1^).

The ground vibrational state potential energy surface contains six equivalent minima corresponding to a single conformer. The contours of Figures 2, 3 represents layers of the 3D-surface of the main isotopologue containing the minimum energy structure. Figure 2 corresponds to V^eff^ (α, β; θ = 270°) and Figure 3 to V^eff^ (α, θ; β = 106°). Figures emphasize the coupling between coordinates.

The kinetic energy parameters were also computed for all the selected geometries and for all the isotopologues. The number of selected geometries required for their computation in the deuterated forms was 171 and 393 for CH_3_NDH and CDH_2_NH_2_, respectively. For all the symmetries, the diagonal terms B_ββ_, B_αα_, and B_θθ_ transform as the totally symmetric representation A_1_. However, the symmetry properties of the off-diagonal elements vary with the MSG:

B_αθ_ transforms as B_1_ (G_12_, G_4_) and A_1_(G_6_)

B_αβ_ transforms as B_2_ (G_12_, G_4_) and A_2_(G_6_)

B_θβ_ transforms as A_2_ (G_12_, G_4_, G_6_)

The non-zero coefficients A_000_(B_qiqz_) of the kinetic energy expressions are shown in Table 4. For the main isotopologue, they are compared with previous data (Ohashi et al., 1988, 1992), although in works based in experiments, these coefficients are considered to be constants. The potential energy barriers, V^tor^ and V^inv^ were estimated using the effective potentials. For the main isotopologue, they are in reasonable good agreement with previous data (Ohashi et al., 1988, 1992) (Kreglewski 1993). Isotopic shifts of all the potential parameters are only important for the deuterated forms.

**TABLE 4:** CCSD(T)-F12/AVTZ potential energy barriers (in cm^−1^).

	CH_3_-NH_2_		^13^CH_3_-NH_2_	CH_3_-^15^NH_2_	CH_3_-NDH	CDH_2_-NH_2_
	*This work*	*Previous works*	*This work*	*This work*	*This work*	*This work*
V^tor^	703	684.71(1)^a^	704	704	692	691
		681.0(5)^b^				
		714.55^c^				
V^inv^	1907	1931.26^c^	1927	1926	1890	1907
A_000_(B_ββ_)	34.8345		34.8021	34.7823	25.9961	35.1168
A_000_(B_αα_)	24.9448		24.9168	24.7859	26.9749	20.3891
A_000_(B_θθ_)	19.0289	15.1130(2)^a^	19.0349	19.1537	19.4989	20.7992
		15.03(1)^b^				
A_000_(B_αθ_)	0.0		0.0	0.0	5.0554	−0.0129

a) Ohashi et al., 1988; b) Ohashi et al., 1992; c) Kreglewski 1993.

Symmetry adapted series were employed as trial functions for the variational calculations. Products of harmonic oscillator solutions X_K_ (for the bending coordinate) and double Fourier series (for the wagging and torsional coordinates) were employed. Table 5 shows the symmetry eigenvectors. The convergence of the low energy levels requires long basis sets leading to Hamiltonian matrices of 18,755 x18,755 elements. In the case of the G_12_ species, the matrices factorize by symmetry into eight blocks which dimensions are 1815 (A_1_, B_2_), 1,518 (B_1_), 1,507 (A_2_), and 3,025 (E_1x_, E_1y_, E_2x_ and E_2y_). For the G_6_ species, the corresponding submatrix dimensions were 3,333 (A_1_), 3,322 (A_2_), and 6,050 (E), whereas for the G_4_ species, the dimensions were 4,840 (A_1_, B_2_), 4,543 (B_1_), and 4,532 (A_2_).

**TABLE 5:** Symmetry eigenvectors.^a^.

**G_12_**	
**A_1_**	**E_1x_**
X_K_ cos (Lα) cos6Mθ	X_K_ cos (Lα) cos (6M ± 1)θ
X_K_ sin (Lα) sin(6M + 3)θ	X_K_ sin (Lα) sin (6M ± 2)θ
**B_1_**	**E_1y_**
X_K_ cos (Lα) cos(6M + 3)θ	X_K_ sin (Lα) cos (6M ± 2)θ
X_K_ sin (Lα) sin6Mθ	X_K_ cos (Lα) sin (6M ± 1)θ
**B_2_**	**E_2x_**
X_K_ sin (Lα) cos6Mθ	X_K_ cos (Lα) cos (6M ± 2)θ
X_K_ cos (Lα) sin(6M + 3)θ	X_K_ sin (Lα) sin (6M ± 1)θ
**A_2_**	**E_2y_**
X_K_ sin (Lα) cos(6M + 3)θ	X_K_ sin (Lα) cos (6M ± 1)θ
X_K_ cos (Lα) sin6Mθ	X_K_ cos (Lα) sin (6M ± 2)θ
**G**_**6**_	
**A1**	**Ex**
X_K_ cos (Lα) cos3Mθ	X_K_ cos (Lα) cos(3M ± 1)θ
X_K_ sin (Lα) sin3Mθ	X_K_ sin (Lα) sin(3M ± 1)θ
**A2**	**Ey**
X_K_ sin (Lα) cos3Mθ	X_K_ sin (Lα) cos(3M ± 1)θ
X_K_ cos (Lα) sin3Mθ	X_K_ cos (Lα) sin(3M ± 1)θ
**G**_**4**_	
**A_1_**	**B_2_**
X_K_ cos (Lα) cos2Mθ	X_K_ sin (Lα) cos2Mθ
X_K_ sin (Lα) sin(2M + 1)θ	X_K_ cos (Lα) sin(2M + 1)θ
**B_1_**	**A_2_**
X_K_ cos (Lα) cos(2M + 1)θ	X_K_ sin (Lα) cos(2M + 1)θ
X_K_ sin (Lα) sin2Mθ	X_K_ cos (Lα) sin2Mθ

a) K, L, M = 0, 1, 2, 3,...

The resulting energy levels are shown in Table 6 and they are classified using symmetry and the v_7_, v_9_ and v_15_ quantum numbers. For the main isotopologue, the energies are compared with those of Kreglewski (1989) obtained from experimental parameters. The computed levels denote a slight improvement with respect to the work of Senent (2018), after using longer expansions for the kinetic energy parameters. The aim was to increase precision considering that isotopic shifts are relatively small. We observed that the vibrational energies are very sensitive to the kinetic contributions. It can be pointed out that their computations in the deuterated forms is not straightforward.

**TABLE 6:** CCSD(T)-F12 energy levels corresponding to the large amplitude vibration and to the HNH bending mode (in cm^−1^). For the main isotopologue, the energies compared with previous data obtained using a two-dimensional model.

**ʋ_NN=7,9,15_**		**CH_3_NH_2_ (G_12_)**		**^13^CH_3_-NH_2_ (G_12_)**	**CH_3_-^15^NH_2_ (G_12_)**	**CH_3_-NDH (G_6_)**		**CDH_2_-NH_2_ (G_4_)**	
		***This work***	**Kreglewski (1989)**			***This work***			
	*3D*	*2D*			*3D*			
0 0 0	A_1_	0.000	0.000	0.000	0.000	A_1_	0.000	A_1_	0.000
	B_2_	0.163	0.078	0.167	0.153	A_2_	0.071	B_2_	0.015
	E_1_	0.325	0.283	0.328	0.322	E	0.117	A_1_	1.491
	E_2_	0.407	0.338	0.412	0.398	E	0.167	B_2_	1.542
								B_1_	1.605
								A_2_	1.672
0 0 1	B_1_	265.572	269.88	264.441	264.428	A_1_	236.260	A_2_	254.110
	A_2_	266.117	270.20	264.995	264.936	A_2_	236.470	A_1_	254.315
	E_1_	259.316	260.94	258.241	258.260	E	233.470	B_1_	254.384
	E_2_	259.066	261.18	257.987	258.027	E	233.571	B_2_	254.589
								B_1_	259.182
								A_2_	259.774
0 0 2	A_1_	447.074	419.47	446.642	447.022	A_1_	416.671	B_2_	436.260
	B_2_	447.418	420.17	446.981	447.347	A_2_	416.888	A_1_	436.350
	E_1_	484.483	464.93	485.819	485.980	E	439.614	B_2_	436.868
	E_2_	486.661	464.36	485.990	486.150	E	439.790	B_1+_	469.827
								A_2_	470.213
								A_1_	470.501
0 1 0	A_1_	771.083	729.39	776.413	763.689	A_1_	715.178	B_2_	767.484
	B_2_	775.011	727.36	777.617	769.602	A_2_	718.848	A_1_	767.952
	E_1_	773.482	769.96	778.819	765.948	E	715.901	B_1_	772.529
	E_2_	777.407	766.97	782.846	769.669	E	718.291	A_1_	774.561
								A_2_	775.594
								B_2_	779.235
0 0 3	B_1_	764.073	732.43	763.519	763.290	A_1_	664.079	A_1_	598.372
	A_2_	764.112	733.47	763.543	763.344	A_2_	664.202	B_2_	598.567
	E_1_	623.966	586.69	623.904	624.147	E	566.925	A_2_	599.434
	E_2_	623.909	587.55	623.863	624.088	E	567.015	B_1_	599.613
								B_1_	724.376
								A_2_	724.454
0 0 4	A_1_	779.811	776.16	779.725	779.716	A_1_	689.459	B_2_	737.897
	B_2_	783.640	783.91	786.487	781.134	A_2_	689.207	A_1_	739.049
	E_1_	961.292	917.30	960.940	960.471	E	825.383	A_1_	905.008
	E_2_	960.857	919.24	960.579	959.974	E	825.524	B_2_	905.345
								A_2_	905.499
								B_1_	905.780
0 1 1	B_1_	1,013.573	1,018.95	1,017.495	1,005.532	A_1_	935.768	A_2_	997.758
	A_2_	1,036.858	1,038.29	1,041.180	1,027.452	A_2_	945.547	B_1_	1,000.853
	E_1_	1,018.895	1,008.94	1,023.138	1,010.215	E	933.720	A_1_	1,001.672
	E_2_	1,009.382	1,010.33	1,013.343	1,001.258	E	938.159	B_1_	1,008.420
								B_2_	1,011.166
								A_2_	1,026.022
0 1 2	A_1_	1,164.413		1,169.282	1,157.813	A_1_	1,091.240	A_1_	1,147.762
	B_2_	1,178.154		1,182.743	1,171.147	A_2_	1,102.316	B_2_	1,164.196
	E_1_	1,183.366		1,182.979	1,181.927	E	1,122.545	B_2_	1,195.131
	E_2_	1,183.676		1,183.288	1,182.336	E	1,131.506	B_1_	1,195.903
								A_1_	1,202.220
								A_2_	1,207.561
0 2 0	A_1_	1,383.962		1,389.035	1,372.966	A_1_	1,255.006	A_1_	1,382.358
	B_2_	1,423.217		1,427.359	1,414.927	A_2_	1,271.686	B_2_	1,393.998
	E_1_	1,408.533		1,414.258	1,398.004	E	1,267.221	B_1_	1,404.544
	E_2_	1,443.195		1,448.522	1,431.945	E	1,280.415	B_2_	1,409.878
								A_1_	1,431.111
								A_2_	1,432.783
									
1 0 0	A_1_	1,648.554	1,628.67	1,636.359	1,656.933	A_1_	1,407.586	A_1_	1,643.044
	B_2_	1,659.193	1,651.28	1,648.221	1,667.223	A_2_	1,436.635	A_2_	1,643.704
	E_1_	1,637.115		1,629.309	1,679.237	E	1,404.920	A_1_	1,649.293
	E_2_	1,656.584		1,646.585	1,662.688	E	1,418.014	B_2_	1,661.305
								B_2_	1,670.498
								B_1_	1,673.893
ZPVE		1,382.996	561.00	1,381.794	1,383.940	1,191.310		1,370.190	

**FIGURE 4:**
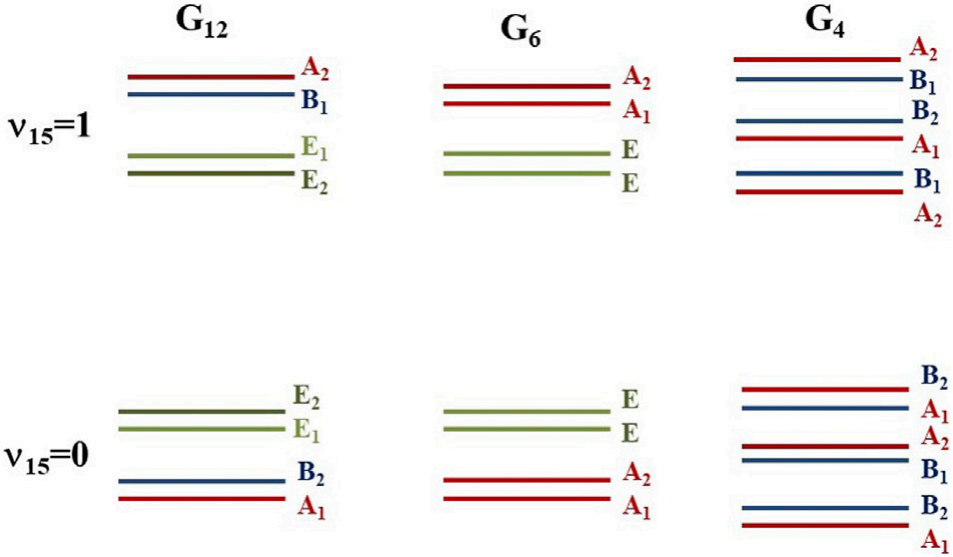
Subcomponents of the (0 0 0) and (0 01) energy levels.

Each energy level splits into six components corresponding to the six minima of the potential energy surface. Their distributions are represented in Figure 4. In the G_12_ species, the levels split into two non-degenerate and two double-degenerated sublevels. The components of the ground vibrational state were computed to lie at 0.000 (A_1_), 0.163 (B_2_), 0.325 (E_1_), and 0.407 (E_2_) cm^−1^. Very small shifts are found for ^13^CH_3_-NH_2_, whereas for CH_3_-^15^NH_2_, the subcomponents are close in energy (0.000 (A_1_), 0.153 (B_2_), 0.322 (E_1_), and 0.398 (E_2_)). The non-degenerated components B_1_ and A_2_ of the ʋ_15_ fundamental (0 0 1) were obtained to lie at 265.572 and 266.117 cm^−1^ in the main isotopologue and at 264.441 and 264.995 cm−1 in ^13^CH_3_-NH_2_, and at 264.428 and 264.936 cm^−1^ in CH_3_-^15^NH_2._ For ʋ_9,_ the corresponding components of the (0 1 0) level were obtained to lie at 771.083 and 775.011 in the main isotopologue and at 776.413 and 777.617 cm−1 in ^13^CH_3_-NH_2_, and at 763.413 and 769.602 cm−1 in CH_3_-^15^NH_2._ It may be concluded that the effects of isotopic substitutions on the heavy atoms are less relevant for the torsional excitation than for inversion excitations.

As was expected, isotopic effects on the low-lying energies are more noticeable for the deuterated species. For CH_3_-NDH, the nondegenerate components of the ʋ_9_ and ʋ_15_ fundamentals have been computed to be 236.260 and 236.470 cm^−1^, and to be 715.178 and 718.848 cm^−1^. The gaps among subcomponents of the ground vibrational state are smaller than in the hydrogenated species. The isotopic substitution in one methyl group hydrogen breaks ten the degeneracy of the CDH_2_NH_2_ levels. The ground vibrational state splits into two A_1_, two B_2_, one B_1_ and one A_2_ components lying in the 0.000–1.672 cm^−1^ range.

## Conclusion

This work describes the shifts of spectroscopic parameters and the symmetry changes due to the isotopic substitutions for various probably detectable methylamine isotopologues, ^13^CH_3_NH_2_, CH_3_^15^NH_2_, CH_3_NHD, and CDH_2_ND_2_. A variational procedure and VPT2 theory are employed for describing rovibrational properties with a special attention to the far infrared region. For all the isotopologues, the levels up to 1,500 cm^−1^ over the ground vibrational state are determine variationally and classified using the G_12_, G_6_ and G_4_ MSG properties. For the main isotopologue, the ground vibrational state splits into six components computed to lie at 0.000 (A_1_), 0.163 (B_2_), 0.325 (E_1_), and 0.407 (E_2_) cm^−1^. Very small differences are found for ^13^CH_3_-NH_2_, whereas for CH_3_-^15^NH_2_, the computed subcomponents are close in energy (0.000 (A_1_), 0.153 (B_2_), 0.322 (E_1_), and 0.398 (E_2_)). Isotopic shifts are relevant for the deuterated forms, whereas the effects of substitution of heavy atoms are less relevant for the torsional excitation than for inversion excitations. Small variations of the kinetic energy parameters carry out substantial displacements of the levels. It can be pointed out that their computations in the deuterated forms is not straightforward.

## Data Availability

The original contributions presented in the study are included in the article/Supplementary Material, further inquiries can be directed to the corresponding authors.
